# HEALTH LITERACY, MEDICAL NON-ADHERENCE, AND SELF-REPORTED HEALTH PROBLEMS AMONG OLDER INTERNET USERS

**DOI:** 10.1093/geroni/igz038.1187

**Published:** 2019-11-08

**Authors:** Gul Seckin

**Affiliations:** University of North Texas, Denton, Texas, United States

## Abstract

The Internet presents new options for the elderly to gather information to support their health care. Health information gathering is among the major motivations for using the Internet among aging baby-boomers. However, insufficient e-health literacy presents challenges for the aging baby boomers. We examined the extent to which health-related internet use and e-health literacy are associated with non-adherence and self-reported negative health outcomes. Respondents were randomly sampled from the largest national online probability-based research panel (N = 710; M= 48.8, SD= 16.4). The age range in our research allowed us to examine the hypothesized associations across the full sample while focusing on older adults (age ≥ 60; N = 194). Older adults with greater e-health literacy reported higher averages for non-compliance because of information obtained from the Internet [(t (194) = 5.06, p ≤ .0001]. Ordinary least squares regression analyses showed that older adults who reported greater averages on health-related internet use reported higher averages on self-reported health problems (β = .292, p ≤ .01). However, women reported fewer health problems (β = -.217, p ≤ .01). Non-adherence with doctor recommendations is a significant positive predictor of self-reported health problem in the full sample (β = .244, p ≤ .0001) but not among older respondents (β = .032, p ≤ .061). Older individuals will make better utilization of the Internet if health professionals guide them to credible sources for health-related information. Empowerment of individuals to utilize the Internet in an informed manner requires addressing their needs for e-health literacy skills.

